# How Mendel’s Interest in Inheritance Grew out of Plant Improvement

**DOI:** 10.1534/genetics.118.300916

**Published:** 2018-10-01

**Authors:** Peter J. van Dijk, Franz J. Weissing, T. H. Noel Ellis

**Affiliations:** * Keygene N.V., 6708 PW Wageningen, The Netherlands; † University of Groningen, 9747 AG, The Netherlands; ‡ Netherlands Institute for Advanced Study, 1012 CG Amsterdam, The Netherlands; § School of Biological Sciences, University of Auckland, 1142 New Zealand

**Keywords:** Gregor Mendel, genetics, inheritance, plant breeding, horticulture

## Abstract

Despite the fact that Gregor Mendel is generally respected as the founder of genetics, little is known about the origin of and motivation for his revolutionary work. No primary sources are known that discuss his work during the period of his pea crossing experiments. Here, we report on two previously unknown interconnected local newspaper articles about Mendel’s work that predate his famous *Pisum* lectures by 4 years. These articles describe Mendel as a plant breeder and a horticulturist. We argue that Mendel’s initial interests concerned crop improvement, but that with time he became more interested in fundamental questions about inheritance, fertilization, and natural hybridization.

## Abstract (precis)

Gregor Mendel’s crossing experiments in pea are the foundation of classical genetics, but since the importance of his 1866 paper was not understood until after long after his notebooks were burned, we know little...

GREGOR Mendel (1822–1884) is recognized as the founder of genetics because of the garden pea and common bean crossing experiments described in his famous article “Experiments on Plant Hybrids” (1866). Although this paper is now > 150 years old, it is still intensively studied. Recently, new English translations have appeared: a Darwinian interpretation (Abbott and Fairbanks 2016; Fairbanks and Abbott 2016) and one with scholarly annotations by the British Society for the History of Science (Mendel 2016).

During his life, Mendel’s work was not appreciated and his notes were destroyed after his death, so when his work came to light in 1900, there were few primary historical sources left and therefore relatively little was known about his biological work and reasoning. While Mendel’s experiments and insights are treated as foundational in virtually all textbooks of genetics, Mendel as a scientist remains a rather mysterious figure. In fact, the major Mendel biographies provide hardly any information about his work in the crucial period of 10 years (1854–1863) during which Mendel performed his famous crossing experiments with peas (Iltis 1924; Richter 1943; Olby 1985; Weiling 1991; Orel 1996). The lack of primary sources has led to a plethora of speculation about Mendel’s intentions and standpoints, aptly described by Sapp (1990) as “the nine lives of Gregor Mendel.”

Most of these views share the “orthodox interpretation” that Mendel was trying to understand the rules of inheritance (Hartl and Orel 1992). In striking contrast, the “revisionist interpretation” claims that Mendel was primarily interested in the question of whether new species could arise from hybridization (Brannigan 1979; Olby 1979; Callender 1988), and that “the laws of inheritance were only of concern to him in so far as they bore on his analysis of the evolutionary role of hybrids” (Olby 1985). According to Olby (1979) the orthodox view is a “whiggish” *post hoc* interpretation that is not in line with Mendel’s original intentions. Olby (1997) argues that it would be “odd” that the terms “heredity,” “hereditary transmission,” and “laws of heredity” do not appear in the title of Mendel’s paper, if Mendel was primarily interested in “searching for the laws of the transmission of characters.” The revisionist view places Mendel in the tradition of earlier hybridizers, notably Josef Gottlieb Kölreuter (1733–1806) and Carl Friedrich Gärtner (1772–1850), and does not recognize Mendel’s work as being revolutionary. Here, we report on two rediscovered newspaper articles that shed new light on Mendel’s motivations to carry out his hybridization experiments.

## Primary Historical Sources About Gregor Mendel

We have several kinds of primary sources concerning Mendel’s work. First, there are Mendel’s scientific papers. “Experiments on Plant Hybrids” is 44 pages long, and contains a short introduction and a long discussion that touches upon diverse topics. In addition, Mendel published a brief paper on artificial hybridization in *Hieracium* (hawkweeds) (Mendel 1870) and two short papers about pests in crops (Mendel 1853, 1854). This is a meager record of 2 decades of work that eventually caused a major scientific transformation. Scientific papers are not diaries, but rather retrospective reconstructions that often misrepresent the history of the research (Sapp 1990), so we only have access to Mendel’s rationalization and description after he had reached his conclusions rather than his thought processes during his work. This is illustrated by the fact that Mendel’s paper “Experiments on Plant Hybrids” was strongly influenced by Darwin’s “Origin of Species,” as shown by Fairbanks and Abbott (2016), but (a German translation of) Darwin’s book became available to Mendel only at the end of his *Pisum* experiments (Darwin 1863) and can therefore not have affected Mendel’s thinking when designing and conducting his experiments.

A second primary source are Mendel’s letters. Letters are more like diaries and often describe thoughts and motivations. Some of Mendel’s correspondence with Carl Nägeli, professor of botany in Munich, is known, including 10 letters that were published by Correns (1905). These letters are mainly about his *Hieracium* experiments, which took place in the 8 years following his *Pisum* work (1866–1873). Furthermore, 18 letters that Mendel wrote to his relatives and friends are preserved (Kříženeckӯ 1965), but in these he did not write about his experimental work. The size and content of Mendel’s correspondence is in striking contrast to that of Charles Darwin, the other 19th century giant of biology, whose correspondence consists of > 15,000 surviving letters and which provides us with a deep insight in his thinking (https://www.darwinproject.ac.uk/letters/darwins-life-letters).

A third primary source are the first-hand accounts of people who met Mendel during his life. In the first decade after the rediscovery of Mendel’s work, many of his pupils from the secondary school were still alive. Iltis (1924, 1966) quotes several of them. Like the recollections of his friend Gustav Niessl von Mayendorf (1839–1919), the secretary of the Natural Science Society, they are mainly anecdotal (Kříženeckӯ 1965). The only professional information is given by Eichling (1942), who visited Mendel in 1878 as a young salesman for a seed company; with whom, however, Mendel was reluctant to enter into a discussion of his pea experiments.

Finally, a fourth source are newspapers from Mendel’s time. Two short reports in the local newspaper *Neuigkeiten* about the *Pisum* lectures Mendel gave in February and March 1865 were discovered by Sajner (1966). Recently, Zhang *et al.* (2017) have reinterpreted Mendel’s pea experiments based on these two newspaper articles. In the last decade, many old newspapers have become available online and these represent a unique searchable source for primary information. Recently, one of us (P. J. van Dijk) found two newspaper articles about Mendel and his work that have not previously been referred to. Mendel is also mentioned in a number of smaller, more general newspaper articles. From these, we have extracted a number of quotations to give an idea of how his fellow citizens thought about Mendel and his work. The digital libraries that were consulted are given in the Supplemental Material.

## Two Rediscovered Newspaper Articles About Mendel’s Work

**Figure 1:**
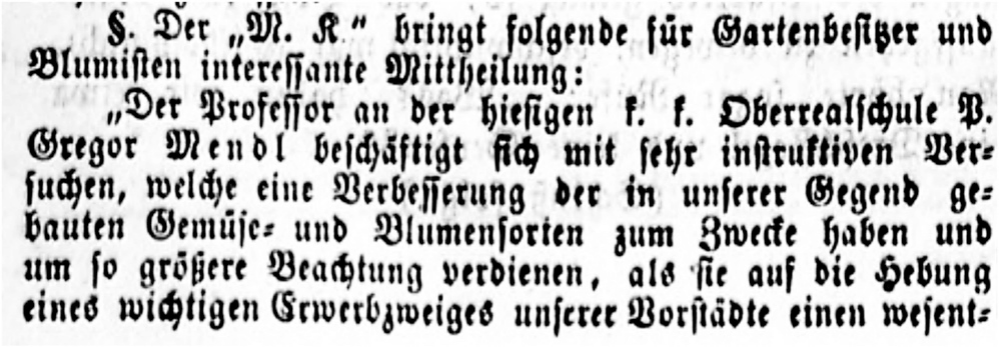
The opening of the newspaper article in *Neuigkeiten* of 26 July 1861 (Anonymous 1861a). Courtesy of DIFMOE (Digitales Forum Mittel- und Osteuropa, München).

The first article (Figure 1, for the original German text see the supplemental material, Anonymous 1861a) appeared on July 26, 1861 in “*Neuigkeiten*” (“News”), a daily newspaper of Brünn, the capital of Moravia, where Mendel lived (now Brno, Czech Republic). It is a copy of an article in *Mährischer Korrespondent (M. K.)* (Moravian Correspondent), another newspaper in Brünn and its surroundings. The Brünn newspapers were printed in German gothic fonts, which are often not well converted by Optical Character Recognition (OCR) software. A search for “Mendel” or “Mendl” did not find the article, but a search for “*Befruchtung*” (fertilization) did. The OCR reads Mendl the first time as “Wenol” and the second time as “Mc »dl,” explaining why the first searches were fruitless. The article may not have been found in the paper versions in the past, because it does not have a separate headline but is part of a feature “*Brünner Zuschauer*” (Brünner Spectator). The corrected and translated text reads as follows:

The “M. K.” brings the following interesting information for garden owners and Brünn: Father Gregor Mendl, professor at the local k.k. Oberrealschule, is concerned with very instructive experiments, which are aimed at improving the vegetable and flower varieties cultivated in our region. They deserve more attention because they should be able to exert a considerable influence on the raising of a vital economic activity in our suburbs. Through artificial fertilization truly surprising results could be achieved. The vegetables grown by the professor, such as peas, fisols^2^, cucumbers, and beans, are high towering bushes that are distinguished by a massive production of fruit which, in size and taste, leave nothing to be desired. For the cultivation of these plants mainly seeds from abroad were used. Of the foreign vegetables so far the New Zealand spinach, which thrives in our soil, was acclimatized. The very fleshy leaves not only contain more nutritious substances than the now cultivated varieties, but the plant is characterized by luxuriant growth, so that some specimens cover their rather large experimental plot almost entirely with their leaves. Until now, the experiments carried out with potatoes were less successful. The plants showed a very vigorous development, but the fruits^3^ started to rot and so far no remedy has been found. Professor Mendl has temporarily extended his experiments [also] to several species of flowers, which up to now have had to be imported at great expense from abroad. The carnations and fuchsias, of which the Professor grew several 100 pots, stand out by their abundance and colorful splendor in an astonishing way. Considering the efforts and diligence which these experiments require to obtain a successful result, one must give all recognition to the professor’s endeavor. The substantial amounts of money that are currently spent on buying seed abroad can better be preserved for domestic production (Anonymous 1861a).

This article shows appreciation and admiration for Mendel’s work. Interestingly, the article provoked a reaction 4 days later, July 30, 1861, in the *Brünner Zeitung* (“Brünner Newspaper”), another local newspaper, arguing that the reporter had overstated the importance of Mendel’s work for the local economy (for the original German text see supplemental material, Anonymous 1861b):

Brünn - We read in a local newspaper an article, which is supposed to be interesting for gardeners and florists, on the acclimatization experiments of Prof. Mendl, in the Augustinian Community of St. Thomas Monastery at Altbrünn. Without wanting to offend Professor Mendl, for we honor every endeavor to approach truth in a practical manner, we must make our readers aware of the true value of the matter, which the reporter has somewhat exaggerated.

Concerning the cultivation of New Zealand spinach *Tetragonia expansa* Murr; its cultivation and use as a vegetable is not new because it was already introduced from New Zealand into Europe in 1772. Mister Schebanek, head gardener of the city of Brünn, has cultivated it for several years in the small plots near the greenhouses, like we ourselves. Despite many years of cultivation, it has not become popular because it has a bitter aftertaste. More common, because more appreciated, is the perennial winter spinach, *Rumex patientia* L., which came in 1573 from Italy to England and Germany; this has a more acid taste and when mixed with common spinach masks the grassy taste of the latter. The common spinach *Spinacia oleracea* L. is dioecious like hemp, which means that some plants only have male flowers and other plants only have female flowers, and only the latter produce seeds for sowing. It arrived here in 1568 from Arabia. In addition to these plants there are others that can be used as spinach, for example red orach *Atriplex purpurea* L. from central Asia, grows in the wild and always has been an appreciated leafy vegetable. The ice plant *Mesembryanthemum crystallinum* L. came from Cape of Good Hope to England in 1727 a.s.o.

Concerning the bastardization of beans, peas or fisols and cucurbits; the seed catalogs from France, England and Germany list so many varieties of excellent quality that it is hardly noteworthy to mention the economic importance of these very small-scale experiments.

Bastardization or hybridization (the transfer of pollen to the stigma of another plant with a fine brush; in most cases the thus produced seeds are of a novel variety) of carnations and fuchsias is an old and generally known practice, currently well known to every thinking garden assistant. The first, the scarlet fuchsia, *Fuchsia coccinea* Art. was brought from Chile to England by a ship captain in 1788. The next was the slender *F. gracilis*, from Mexico in 1822 and then the small-leaved *F. microphylla* in 1827. Up to now there are 32 constant species which all originate from South America and some 500 hybrids which have been produced in Europe. This beautiful and easy to cultivate plant group has been named Fuchsia to honor Leonhard Fuchs who died as Prof. in Medicine in 1565 in Tübingen. He was a botanist and defender of Hippocratic medicine and was made a nobleman by Charles V. This genus was also named *Nahusia* Schoe.; *Skinnera* Mönch.; *Quelusia* Vand. but only the name after Fuchs persisted (Anonymous 1861b).

This second article is rather negative about Mendel’s horticultural work, although it purports to recognize its scientific value: “we honor every endeavor to approach truth in a practical manner.” This remark is intriguing, since it suggests that Mendel was empirically solving a scientific question, which in this instance would have been the understanding of the inheritance of traits.

These two new articles date from the period in which Mendel was in the middle of his *Pisum* experiments, and are the only significant sources so far about his work between the finishing of his University studies at Vienna in July 1853 and the two pea lectures in February and March 1865. The articles in local newspapers reflect the interest from the general public for the type of work that Mendel was doing. These newspapers were widely read and together had a daily circulation of > 6000 copies (Anonymous 1875); at that time, Brünn had a population of 70,000 (Eichling 1942). An 1873 article in *Tagesbote*^4^ called Mendel “a widely known zealous horticulturist and pomologist”^5^ (Anonymous 1873). We can thus conclude that Mendel’s horticultural activities were quite well known in Brünn at the time.

## The Newspaper Articles in Relation to Mendel’s *Pisum* Paper

Mendel conducted his pea crossing experiments between 1856 and 1863 (see Mendel’s second letter to Nägeli; Correns 1905). Before that, in 1854 and 1855, he tested the material for true breeding traits (Mendel 1866). In his second letter to Nägeli (April 1867), Mendel described a special pea variety that he had bred and cultivated, and that was not mentioned in the 1866 paper: “I must further mention the case of a [pea] variety which bred true for six generations, although the parental types differed in four characters. In 1859 I obtained a very fertile descendant with large, tasty, seeds from a first generation hybrid [nowadays the F2]. Since, in the following year, its progeny retained the desirable characteristics and were uniform, the variety was cultivated in our vegetable garden, and many plants were raised every year up to 1865.” (p5, Piternick and Piternick 1950). This may have been one of the varieties that is mentioned in the first newspaper article. Because the homozygous F2 was obtained in 1859, the parents must have been crossed no later than 1857. Probably this same variety was still grown 11 years later when, in the summer of 1878, Eichling visited Mendel: “Mendel had imported over 25 varieties of peas, which shelled out readily, but did not yield very well because some of them were bush types. As I recall it, he said that he crossed these with his tall local sugar-pod types and now had tall shelling types, which were used at the monastery.” (Eichling 1942). Both Eichling and the first newspaper article state that Mendel had used mainly seeds from abroad.

It is notable that the first article states that Mendel, apart from his work on peas and beans, also carried out artificial crosses with cucumbers. Mendel’s gardener, Joseph Maresch, told Hugo Iltis of Mendel’s work on cucumber half a century later, but Iltis thought his memory was unreliable because of his bad drinking habits (Iltis 1924).

**Figure 2:**
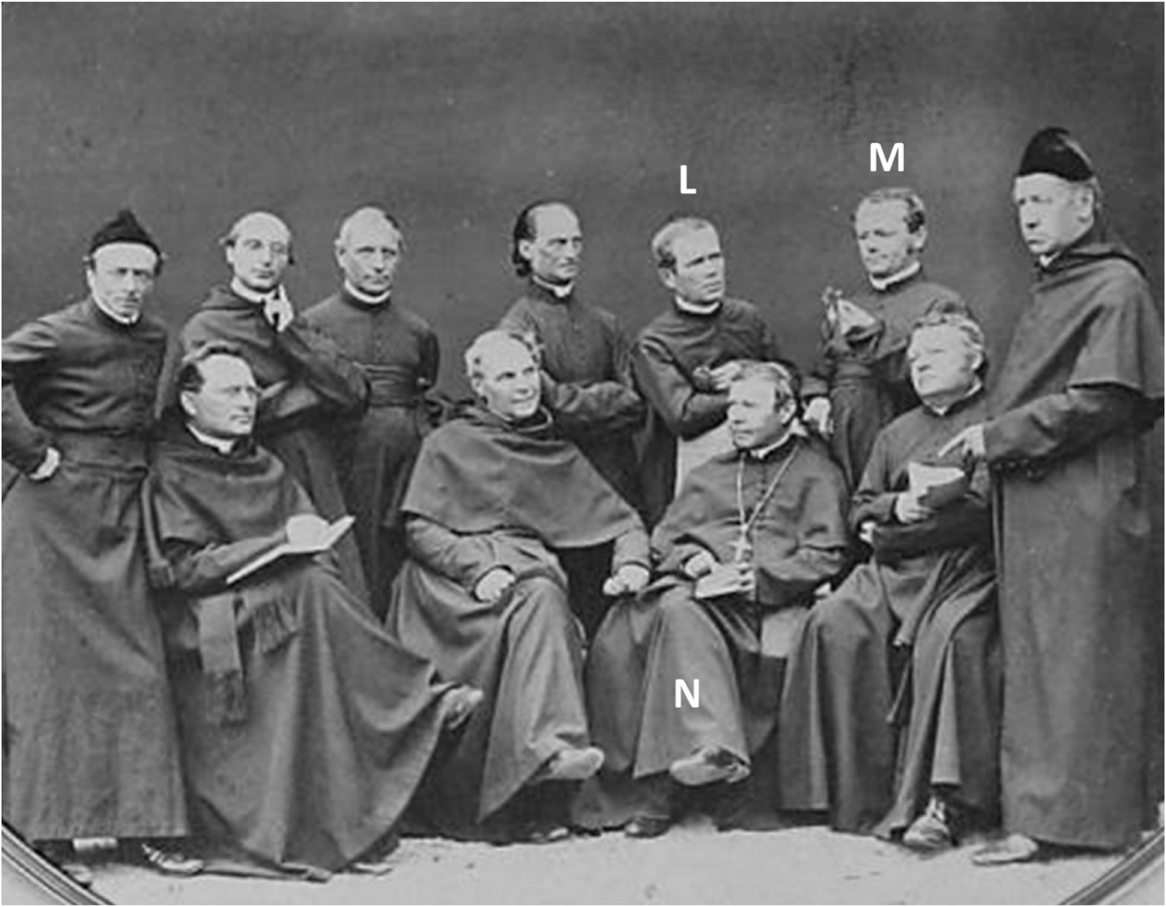
A photograph of the brothers the Augustinian Community of St Thomas Monastery in Brünn ∼1862. Abbot Cyrill Napp (N) is seated with the pectoral cross and bible, wearing a pileolus. He is flanked by Gregor Mendel (M) and Joseph Lindenthal (L), both leaning on his chair. Lindenthal helped Mendel with his crossing experiments (Iltis 1924). Both hold a flower in their hand. From the symbolism of the objects associated with each person, it is clear that the photo is arranged and it is probably no coincidence that Napp, with his interest in inheritance, is flanked by the two plant breeders. Courtesy of the Mendel Museum of Masaryk University, Brno, Czech Republic.

Mendel began the 1866 paper with: “Artificial fertilizations that were carried out on ornamental plants with the aim of producing new color variants, provided the motivation for the experiments that shall be reviewed here,” leading Muller-Wille and Hall (Mendel 2016) to comment: “It is not clear to which experiments Mendel is referring here.” The first newspaper article mentions that Mendel had extended his experiments to carnations (*Dianthus*) and fuchsia plants. These crosses with *Dianthus* and *Fuchsia* can be seen as the motivation and inspiration that Mendel speaks of at the beginning of the 1866 paper. Carnation experiments are mentioned in the 1866 article and Mendel’s interest in fuchsia was known. In two group photos of that time (∼1862), he stands among the other brothers of the Augustinian Community of St. Thomas Monastery in Brünn with a fuchsia flower in hand (Figure 2). The first newspaper article says that Mendel had several hundred pots of fuchsia, which would have overwintered in his heated greenhouse. Mendel was acquainted with the famous fuchsia breeder J. Twrdy (1806–1883), who named a new variety after him (*Prälat Mendel*) (Orel 1996).

## Mendel the Horticulturist

The first newspaper article furthermore shows that Mendel was also occupied in the early 1860s with the introduction of new crops and the control of plant diseases; Mendel’s growth experiments with spinach and potato are unknown. Mendel’s interest in potato disease is in line with his two earlier publications about harmful insect pests of radish and peas, respectively. Potato blight, caused by the fungus *Phytophtera infestans*, was a major problem throughout Europe in the middle of the 19th century, and reports on “*Kartoffelfäule*” regularly appear in the Brünn newspapers of those days. In one of the surviving letters to his parents, dated December 28, 1851, Mendel writes that to his dismay he heard that the potato disease had spread to the region where his parents lived (Kříženeckӯ 1965):

Köningskloster am 28 Dec. [1851?]^6^.

Dearest parents!

I have heard with dismay that the potato disease is also spreading in your region. Almost throughout the whole of northern and central Europe, this disease has caused much damage in the fields and in the cellars. In any case, the previous wet years are to blame. From day to day the deprivation increases among the poorer people as with ever increasing grain prices, also potatoes become unaffordable.

Treatments against the rot will have been made known to you by the authorities. The best thing to do is to separate the healthy from the rotten ones. The former should be dried and stored in dry places. The latter however, should be spread out and desiccated to prevent further rotting to use it at least as cattle feed.

Your always thankful son (Kříženeckӯ 1965).

Mendel may have been trying to obtain resistance by crossing potato species or varieties, since Friedrich Klotzsch had obtained resistance by crossing *Solanum utile* with *S. tuberosum* in 1850, producing a “*Bastard-Zuckerkartoffel*.” Klotzsch’s experiments were mentioned in a local newspaper (Anonymous 1855) and in the Proceedings of the Brünn Agricultural Society (Anonymous 1858), so Mendel may have been familiar with them.

## Cyrill Napp, the Link Between Mendel’s Work and the Sheep Breeders Society

The newspaper articles describe Mendel as a horticulturist and plant breeder, improving varieties by artificial fertilization. Where did his interest in plant improvement come from?

Wood and Orel (2005) have suggested that the Abbot Cyrill Napp (1792–1867) had a strong influence. It was Abbot Napp who adopted Johann Mendel as a novice in the St Thomas monastery in October 1843. In the group photo (Figure 2), Mendel stands behind the sitting Napp who facilitated Mendel’s work and further development; he dismissed Mendel from pastoral duties so that he could work as a teacher and arranged for his studies in Vienna. In 1855, Napp had a heated greenhouse built for Mendel’s experiments. There can be no doubt that Napp was aware of Mendel’s experiments. After all, the experiments were conducted in the prelate’s garden, not far from the refectory where the friars had been eating that tasty pea variety bred by Mendel [F. Weiling, cited by Orel (1975)].

In the 1830s and 1840s, Napp was a prominent participant in the discussions of the Sheep Breeders Society, as documented in its minutes. In the first half of the 19th century, Moravia, and more specifically its capital Brünn, was one of the most advanced regions of Europe in breeding (Orel 1996; Orel and Wood 1998; Orel and Czihak 2001; Wood and Orel 2005). Brünn had a large wool industry and was nicknamed Moravian Manchester. The production of high-quality local wool was therefore very important. In 1814, the Sheep Breeders Society at Brünn was established as a section of the Moravian Agricultural Society^7^.

In the annual meeting in 1836, Napp argued that the inheritance of properties from parents to offspring depended primarily on the affinity of the mated animals; therefore, “for each ewe a ram should be chosen that fits best to the internal and external organism of the ewe; this choice needs to be the result of a careful physiological study”^8^ (Anonymous 1836). At the next annual meeting, Napp pointed out that the discussion had completely gone astray from the original topic of hereditary capacity, putting the theory of the breeding process at center stage, when rather this should be the question: “what is inherited and how?”^9^ (Anonymous 1837; Wood and Orel 2005)^10^. Napp thus forms the link between the heredity questions in the 1840s and the nature of inheritance reflected in Mendel’s work in the 1850s and 1860s.

Before Mendel started with his *Pisum* experiments, he had already a considerable knowledge of plant breeding. In 1846, he attended a course in pomology and viticulture given by Professor Franz Diebl, a friend of Abbot Napp, at the Brünn Philosophy Institute. At this course, artificial pollination of plants for the production of new cultivars was taught, both theoretically and practically (Diebl 1844; Orel 1996; Klein and Klein 2013). During his university study at Vienna, Franz Unger was one of Mendel’s botany professors. In his textbooks, Unger (1846, 1855) discusses the transmission of variable traits from mother to daughter plants through seeds, “for example in vegetables and fruit trees, flower varieties (stock (*Matthiola*), carnations, a.s.o.) *etc*.” (Unger 1846, p110). In 1855, Unger included a paragraph entitled “Transmission of the trait of a mother organism to the daughter organism, cross breeding” (Contents section §198 pXVIII). The end of this paragraph reads: “Hybridization has been extensively exploited in horticulture, which has produced a large number of forms of which the parental lines are partly or completely unknown to us.” (p393). Artificial fertilization, transmission of traits, and the application to plant improvement were thus subjects in which Mendel was well trained.

In 1855, Mendel became a member of the Natural Science Section of the Agricultural Society in Brünn. Napp was active in the Pomological Section, suggesting that Mendel’s interests had become purely scientific, possibly because of his university education and reflecting Napp’s earlier call for a physiological study of inheritance. In the year of the newspaper articles (1861), the Natural Sciences Society split from the Agricultural Society, with the aim of better promoting pure science. Mendel switched to the new Natural Sciences Society, while Napp became chairman of the Horticultural Association, which replaced the Pomological Section of the old Agricultural Society. In 1863, Mendel was chosen as a member of the Horticultural Association (Iltis 1924). Later, he became chairman of the judges of the annual horticultural exhibitions and an examiner for fruit-growing courses, reflecting his reputation with respect to horticultural knowledge. In both the Natural Sciences Society and the Horticultural Association, plant hybridization was a recurring theme. According to the minutes of the meeting of the Horticultural Association for October 1864 (while Mendel was conducting his hybridization experiments in the monastery garden), Napp noted that plant hybridization was “still more a question for science than for practice” (Orel 1996). Mendel therefore had interests both in natural science and horticulture.

Mendel held his famous *Pisum* lectures in February and March 1865 at the Natural Science Society and not at the Horticultural Association. Mendel was indeed interested in plant improvement, as reflected by his membership of the Horticultural Association, but his lecture was not given to that society and was instead given to the one with broader scientific interests.

In the report about the first lecture, the *Brünner Morgenpost* wrote: “The relevant observations were mostly made on Papilionaceae (a family that according to well-known researchers is not very suitable for hybridization)”^11^ (Anonymous 1865a), suggesting a concern that this plant material was more appropriate for practical rather than fundamental studies. The members of the Natural Science Society were more interested in natural than in purposeful hybridization.

Taken together, Napp and Mendel covered both the pure and applied science related to breeding, crop improvement, and genetics; it is hard to imagine that they did not talk to each other about this subject. In July of 1867, Abbot Napp died and Mendel was elected as his successor in March 1868.

## Mendel’s Experiments on Hybrids

We can see from these two newspaper articles that Mendel had a background in breeding and note that the breeders (especially of sheep) in Brünn were concerned with how to select the best individuals for (rather than from) crosses. The issue was whether these individuals should be selected because of individual characteristics or an assessment of their overall quality. Mendel had studied combinatorial mathematics in Vienna, under Ettingshausen, so this set of interests may have led him to focus on individual traits in his *Pisum* crossing experiments, rather than on the organism as a whole. This choice was a departure from the practice of previous hybridizers such as Josef Gottlieb Kölreuter and Carl Friedrich Gärtner, as was his choice to study intraspecific hybridization instead of interspecific hybridization.

Mendel will have known that *Pisum* was suitable for the study of individual traits, for example from the work of Thomas Andrew Knight (1759–1838). In his 1866 paper, Mendel gives three reasons for selecting *Pisum*, and these are the reasons given by Knight (1799). In the reference list of his own copy of Gärtner’s masterpiece *Versuche und Beobachtungen über die Bastarderzeugung im Pflanzenreich* (Gärtner 1849), Mendel underlined a reference to Knight’s work (such annotations are another preserved primary source) and Knight’s 1799 paper was available as a German translation in Brünn (Orel 1996). Mendel chose differentiating traits, tested them to be true breeding, crossed the plants, and then counted the different progeny classes and calculated their ratios *etc*. He inferred the purity and composition of the gametes and their random fusion. Zirkle (1951) has suggested that Mendel’s ratio approach may have been inspired by Dzierzon’s 1854 publication of 1:1 segregation of parental types in the drones produced by hybrid queens from matings between Italian yellow and German black bees. The following passage also stresses the importance of starting with true-breeding parents: “One must be absolutely certain that the queen belongs by birth to the pure race. If she herself originates from a hybrid brood, it is impossible for her to produce pure drones, but she produces half Italian and half German drones, but strangely enough, not according to the type [not a half and half intermediate type] but according to number, as if it were difficult for nature to fuse both species into a middle race.” In the same year, 1854, the Apicultural Association of the Agricultural Society was founded to apply Dzierzon’s rational methods of bee keeping, an initiative of Abbot Napp (Orel 1996). In his *Pisum* paper, Mendel developed a theory about the inheritance of traits. He made a clear distinction between “elements” (*Elemente*) and traits (*Merkmale*); these words are used mutually exclusively in different paragraphs in the *Pisum* paper. The elements were nonblending; beyond this property the physical form of the elements was not specified, nor did it need to be.

Pea is an example of a species where hybrids produce variable offspring (variable hybrids), which contrasted with constant hybrids, which produced nonvariable offspring and of which Mendel thought he had found an example in *Hieracium*. Based on detailed analysis of Mendel’s letters to Nägeli, we have recently argued that the traditional interpretation that the *Hieracium* crossing experiments were intended to repeat the *Pisum* experiments, and therefore frustrated Mendel, is wrong (van Dijk and Ellis 2016). With *Hieracium*, Mendel moved to wild species and more clearly to fundamental research. Both peas and hawkweeds were part of his broader research program into different forms of inheritance.

When, long after his death, Mendel’s interpretation of inheritance was rediscovered, it formed the basis for the new field of science baptized by William Bateson as “Genetics” in 1906 (Bateson 1907). However, Mendel’s experiments had more implications, which Mendel discussed in his paper, such as the transformation of one species into another, the cytology of fertilization, the generation of variation by the conditions of life *vs.* hybridization, speciation, and the stability of species and hybrids. All these reflect also Mendel’s interest in pure science. According to the report of the second *Pisum* lecture in the *Mährischer Korrespondent*, Mendel first gave an introduction to (what was known about) “the cell and the reproduction of the plants by fertilization.” before he presented his own research (Anonymous 1865b,c). Therefore, it makes sense that Mendel chose the broad title “Experiments on Plant Hybrids,” without specifically mentioning heredity or inheritance. Mendel’s broad interest in plant biology was clearly sanctioned by Napp’s comments relating to the need for a scientific study of inheritance.

Although the word inheritance was used only once in the text of the *Pisum* paper and was missing from the title, the paper is unmistakably about the rules of inheritance. That was quite clear to Nägeli when he wrote to Mendel: “I am convinced that with many forms you will get notably different results (in respect to the *inherited* characters [our emphasis]).” (Hoppe 1971). However, Nägeli did not believe that Mendel’s elements could be nonblending, as he wrote in his notes: “the constant forms require to be tested further (A, a, AB, Ab, aB, ab – nowadays: *AA*, *aa*, *AABB*, *AAbb*, *aaBB*, *aabb*). I expect that (when inbred) they would sooner or later be found to vary once more. “A” for instance has half “a” in its body [bred out Aa] and when inbred cannot lose that element.” Furthermore, Mendel’s interest in inheritance and its mechanism is clear from his annotations in his own copy of the German translation of Darwin’s *The Variation of Animals and Plants under Domestication* (1868). Most of these annotations can be found in the penultimate chapter of volume 2, dealing with Darwin’s pangenesis theory of inheritance (Orel 1996). That Mendel strongly disagrees with Darwin’s speculative theory is clear by written remarks, like “to succumb to an impression without giving the matter proper thought” (Orel 1996, p194).

In the beginning of the 1870s, Mendel’s *Hieracium* studies became a daunting task. While Mendel had produced *ca*. 200 *Hieracium* hybrids, this had been a huge effort involving many thousands of hand emasculations and cross-fertilizations (van Dijk and Ellis 2016), so he realized that he had to make many more artificial fertilizations to obtain a sufficient number of hybrids to analyze the numerical relationships between the different types or “members of the series.” This was virtually impossible because the emasculation of the tiny flowers had to be done with lens-concentrated light, which already had almost ruined his eyesight.

After 1870, Mendel became very interested in bee breeding and became a member of the Association of Moravian Beekeepers. He had an experimental bee house built in the monastery garden. There, he performed breeding experiments with different bee races. It is tempting to speculate that he was inspired by the discoveries of Dzierzon, but there is no proof to support this idea. According to a report in *Tagesbote* of a meeting of the Beekeepers Association in June 1877, “Prälat Mendel drew attention to the advantages of the Cyprian bee race, especially with regard to its suitability for improvement by breeding”^12^ (Anonymous 1877). Mendel remained a breeder until the end of his life.

Mendel died in January 1884 from heart and kidney failure. The author of the obituary in *Tagesbote* wrote: “Epoch-making were his investigations into plant hybrids”^13^ (Anonymous 1884). This time, the reporter’s prophecy was no exaggeration, although at that time nobody except the deceased understood precisely what his investigations meant.

## Conclusions

The revisionist view, which diminishes Mendel’s role in the development of our understanding of the science of inheritance, is popular among historians and sociologists of science. It is also promoted in popular science books (for example: Bowler 1989; Waller 2002; Endersby 2007; Numbers and Kampourakis 2015; Kampourakis 2016) and its place in the genetics curriculum is being discussed in journals about education (for example: Allchin 2003; Westerlund and Fairbanks 2004; El-Hani 2015; Peterson and Kampourakis 2015). While Olby (1979) rightly points to Mendel’s interest in the origin of species diversity [at least in the constant hybrids (*Hieracium*), much less so in the variable hybrids (*Pisum*)], this does not detract from the possibility of his having other concerns. The newly discovered newspaper articles show that Mendel was actively engaged in plant hybridization for the development of useful varieties, and we know that his first two publications reveal an interest in plant productivity. We fully accept the view that Mendel must be seen in the context of his time and environment. We think that this must take account of Mendel’s immediate environment, especially that of the Augustinian community of St Thomas’ monastery, and notably Abbot Napp who supported Mendel and posed some basic questions about inheritance. In this context, proposing that Mendel was interested in inheritance is not an “inflated whiggish” interpretation (Olby 1979), but grounded in his activities reported at the time and in the profound questions raised by his patron Cyrill Napp.

To summarize, we think that Mendel’s interest evolved from being that of a hybridizer, motivated by practical goals, to that of a scientist motivated by fundamental questions. The basis for this had already been laid during his studies at the university and was clearly catalyzed by his Abbot but, perhaps, also, in 1863–65, by his reading of Darwin.
